# Cardiovascular Risk Factors in Coronary Artery Bypass Graft Patients: Comparison Between Two Periods

**DOI:** 10.7759/cureus.10561

**Published:** 2020-09-21

**Authors:** Anas E Bifari, Rakan K Sulaimani, Yaser S Khojah, Osama S Almaghrabi, Hesham A AlShaikh, Khalid E Al-Ebrahim

**Affiliations:** 1 Medicine, College of Medicine, King Abdulaziz University, Jeddah, SAU; 2 Surgery, King Abdulaziz University, Jeddah, SAU

**Keywords:** coronary artery bypass grafting, risk factors, diabetes mellitus, hypertension, smoking

## Abstract

Background

Information showing risk factor trends in patients undergoing coronary artery bypass graft in Saudi Arabia is scarce. Thus, we aimed to compare cardiovascular risk factors among coronary artery bypass graft patients between two periods: 2012 and 2018.

Methods

This was a cross-sectional study based on hospital records at a tertiary center in Saudi Arabia. The medical records of 72 patients in 2012 and 111 patients in 2018 were reviewed. The study included all patients who underwent coronary artery bypass grafting for the first time. The chi-square test and independent t-test were used for statistical analysis; P-values less than 0.05 were considered statistically significant.

Results

The mean (SD) of the patient age was 61.21 (9.74) years in the first period and 58.01 (11.14) years in the second period. The number of patients who smoked was significantly higher in the second period of the study (14.3% in the first period; 27.0% in the second period; P < 0.001). The study also showed an increase in hypertension and diabetes mellitus in the second period compared to the first (70% vs 71.2% and 68.6% vs 72.1%, respectively), and a reduction in the percentage of patients with hypercholesterolemia (18.3% vs 17.1%). However, these findings were non-significant.

Conclusions

The percentage of smokers was significantly higher in the second period of this research as a consequence of cultural variation and because of the popularity of water-pipe smoking in the society. We recommend the need for increased awareness regarding smoking and the implementation of smoking-cessation programs.

## Introduction

Cardiovascular disease (CVD) refers to a group of illnesses that cause damage to the heart and blood vessels [1]. Over the past years, CVD has been considered a health burden globally [2]. According to a study by the World Health Organization in 2008, CVD was responsible for 7.3 million deaths worldwide [1]. However, in the Gulf region, CVD was reported to be the cause of 45% of all fatalities in the six Gulf Cooperation Council nations. A high percentage of CVD fatalities were recorded in Oman and Kuwait (49% and 46%, respectively), as well as in Saudi Arabia, United Arab Emirates, Bahrain, and Qatar (42%, 38%, 32%, and 23%, respectively) [3-5]. In 2016, 37% of all the deaths in Saudi Arabia were caused by CVD [6]. As a result, over the past decade, coronary artery bypass grafting (CABG) was widely adopted [7-9] and represented 10% of all cardiac procedures in the United States [10].

Risk factors for CVD include hypertension (HTN), dyslipidemia, smoking, diabetes mellitus (DM), obesity, lack of physical activity, and unhealthy diet [11-14], and these factors show variable trends over time [15]. Studies have shown the effect of the presence of multiple risk factors in the same individual, which can increase disability and mortality rates [16]. A study published in 2012 showing the change in risk profile in patients who underwent CABG revealed that there were more patients with DM and fewer smokers in 2009 compared to 2000 [17]. Today, patients undergoing CABG present with more risk factors and comorbidities than in the past [18-20].

A study conducted in Iran found an increase in the prevalence of multiple risk factors in individuals in 2016 compared to 2010 [16], whereas a study published in 2019, which reviewed all patients who underwent CABG in Cape Cod Hospital in the United States between 2000 and 2014, revealed that patients who underwent CABG between 2009 and 2014 were more likely to present with DM [21].

Information showing risk factor trends in patients undergoing CABG in Saudi Arabia is scarce, and it is essential to recognize these trends to increase awareness in our society, which can, in turn, decrease the incidence of CVD.

This study aimed to compare the cardiovascular risk factors among CABG patients between two periods: 2012 and 2018.

## Materials and methods

This was a hospital records based cross-sectional study performed in a tertiary center in Saudi Arabia. The study was conducted from January 2019 and lasted four months. All data were obtained from medical records of health information system (Phoenix) in King Abdulaziz University Hospital (KAUH), a tertiary center in Jeddah, Saudi Arabia, and collected using a data collection sheet. All patients who underwent CABG for the first time in 2012 or 2018 were included; patients who received reoperative CABG were excluded. We reviewed the medical records of 183 patients and collected data that included the diagnoses of DM, HTN, and hypercholesterolemia; tobacco smoking; and a family history of coronary artery disease (CAD). Laboratory results were also collected, including hemoglobin A1c (HbA1c), high-density lipoprotein, low-density lipoprotein, and triglycerides taken on the day of admission. We categorized the patients into three groups: (1) those below 50 years of age, (2) those between 50 and 70 years of age, and (3) those above 70 years of age. Body mass index (BMI) was classified into categories according to guidelines of the Centers for Disease Control and Prevention for screening for overweight and obese patients: (1) underweight, BMI <18.5; (2) normal, BMI from 18.5 to <25; (3) overweight, BMI from 25 to <30; and (4) obese, BMI of 30 kg/m^2^ or higher [22]. Multimorbidity was described as the existence of two or more chronic conditions in one patient. We distributed the patients into four groups based on the existence of CAD plus other morbidities as follows: (1) those with CAD but without other chronic conditions, (2) those with CAD and one chronic condition, (3) those with CAD and two chronic conditions, and (4) those with CAD and three chronic conditions.

The study was approved by the Institutional Review Board of KAUH, and the Ethics Committee did not require patient consent for medical records review. The collected data remained confidential and protected during the study. It was conducted in the Cardiac Surgery Department.

We used IBM SPSS Statistics for Windows (version 21.0, IBM Corp., Armonk, NY, USA) for statistical analysis. The chi-square test was used to assess the relationship between CAD and risk factors, and the independent t-test was computed to compare between both patient groups. A P-value of <0.05 was considered significant.

## Results

We compared CVD risk factors among patients who underwent CABG between 2012 and 2018. The number of CABG patient records reviewed was 183, 72 in the first period and 111 in the second period, accounting for 48.24% and 58.06% of patients, respectively, of patients who underwent cardiac surgery. Most of the patients were men (156 [85.2%]). Patient age ranged from 27 to 88 years, with a mean (SD) of 59.27 (10.7) years. The mean (SD) patient age was 61.21 (9.74) years for the first period and 58.01 (11.14) years for the second (P = 0.048), as shown in Table 1.

**Table 1:** Comparison of risk factors among CABG patients in both periods *Chi-square test; #Independent t-test. SD, standard deviation; BMI, body mass index; CABG, coronary artery bypass grafting

Variables	2012-2013 (n = 72)	2017-2018 (n = 111)	P-value*
Age (year), mean ± SD	61.21 ± 9.74	58.01 ± 11.14	0.048#
BMI (kg/m^2^), mean ± SD	28.37 ± 5.32	26.95 ± 3.93	0.073#
Age category (year), n (%)			<0.135*
<50	10 (13.9)	28 (25.2)	
50-70	50 (69.4)	70 (63.1)	
>70	12 (16.7)	13 (11.7)	
Sex (male), n (%)	60 (83.3)	96 (86.5)	0.708*
Smoking, n (%)	10 (14.3)	30 (27.0)	0.000*
Diabetes, n (%)	48 (68.6)	80 (72.1)	0.737*
Hypertension, n (%)	49 (70.0)	79 (71.2)	0.999*
Hypercholesterolemia, n (%)	13 (18.3)	19 (17.1)	0.960*
BMI categories (kg/m^2^), n (%)			0.027*
Underweight (<18.5)	2 (2.8)	2 (1.8)	
Normal (18.5 to <25)	15 (20.8)	42 (37.8)	
Overweight (25 to <30)	24 (33.3)	40 (36.0)	
Obese (>30)	31 (43.1)	27 (24.3)	
Comorbidity number, n (%)			<0.039*
1	16 (22.2)	11 (9.9)	
2	14 (19.4)	35 (31.5)	
3	30 (41.7)	53 (47.7)	
4	12 (16.7)	12 (10.8)	

Table 1 presents the relationship between smoking and CABG in both periods. There was a significant increase in the number of smokers from 10 in the first period to 30 in the second period (14.3% vs 27.0%; P < 0.001). The number of CABG patients with DM increased from 68.6% in the first period to 72.1% in the second period (P = 0.737). HTN diagnosis increased from 70% in the first period to 71.2% in the second period (P = 0.99). The mean BMI was 28.37 (5.32) kg/m^2^ in the first period and 26.95 (3.94) kg/m^2^ in the second period (P = 0.073), and hypercholesterolemia decreased from 18.3% in the first period to 17.1% in the second period (P = 0.960).

We classified the patients based on age, BMI, and number of comorbidities. Patient distribution according to age showed the following: (1) below 50 (9.7% in the first period; 21.6% in the second period), (2) between 50 and 70 (73.6% and 64%, respectively), and (3) over 70 years (16.7% and 14.4%, respectively). According to BMI, the number of patients in the standard weight group (BMI: 18.5 to <25 kg/m^2^) increased from 20.8% in the first period to 37.8% in the second period, as did the number of patients who were overweight (33.3% to 36%, respectively). According to number of comorbidities, the percentage of patients with CAD and one chronic disease increased from 19.4% in the first period to 31.5% in the second period, and those with CAD and two chronic diseases from 41.7% in the first period to 47.7% in the second period.

As shown in Table 2, a statistically significant relationship was found between CABG patients in both time periods with regard to HbA1c (P = 0.022).

**Table 2:** Laboratory findings of CABG patients in both periods *Independent t-test. SD, standard deviation; Df, degree of freedom; HbA1C, hemoglobin A1c; LDL, low-density lipoprotein; HDL, high-density lipoprotein; TG, triglyceride; CABG, coronary artery bypass grafting

Variables	2012-2013, mean ± SD	2017-2018, mean ± SD	P-value*	Df
HbA1c (mg/dL)	8.86 ± 2.32	7.9 ± 2.29	0.022	1
Cholesterol (mg/dL)	4.43 ± 1.18	4.31 ± 1.51	0.550	1
LDL (mg/dL)	2.87 ± 0.91	2.93 ± 0.17	0.726	1
HDL (mg/dL)	0.92 ± 0.23	0.93 ± 0.20	0.746	1
TG (mg/dL)	1.75 ± 0.87	1.93 ± 1.13	0.255	1

## Discussion

In this study, we aimed to compare risk factor trends among patients undergoing CABG for the first time between 2012 and 2018. A slight decrease in the mean age of CABG patients was found in the second period mainly in patients younger than 50 years. A study conducted in Iran showed similar results, with a reduction in the mean age and increase in CABG patients younger than 50 years in 2016 [23]. This finding may be due to the increased development of the Kingdom of Saudi Arabia (KSA). For example, increasing investments have led to an increase in fast-food restaurant chains and luxury services that have reflected on the citizens’ health (having a more sedentary lifestyle) [24-26].

Our data demonstrate a reduction in mean BMI, with a significant elevation in the normal and overweight BMI groups of CABG patients in the second period. A study found an increase in the percentage of overweight Indian patients undergoing CABG surgery [27]. To explain the increase in the normal BMI group, we assume that the decrease in the obese group and slight increase in the overweight groups led to an increase in the normal group. Furthermore, we believe that the more marked reduction in BMI among CABG patients is due to the effect of social media.

The number of patients who smoked was also elevated in our study, and similar results were noted in studies from China and the United States [17,28]. Despite governmental and individual efforts toward controlling and prohibiting the use of tobacco products and shisha (water-pipe) [29], these are still becoming more prevalent in Arab countries and are considered fashionable among the youth.

An increase in DM and HTN and a slight reduction in hypercholesterolemia were found in our study. Studies conducted in Iran in 2008 and in the United States in 2019 reported similar results [21,30]. These findings may be related to the global increase of DM and HTN considering individuals’ lack of exercise, busy work life, and poor eating habits [6]. The significant increase in comorbidity groups 2 and 3 could be a result of the same reasons mentioned. 

According to our data, a significant decrease in the level of HbA1c was noticed among the CABG patients in 2018. Awareness programs and campaigns in KSA, initiated by the Ministry of Health and implemented by health workers and medical students, have had a beneficial impact on the community and account partially for this result [6].

This study has several limitations. First, it was conducted in a single tertiary center; thus, the sample size was small. Conducting more studies in primary centers should help to verify our findings. Another limitation is the two separate periods we used, as we could include only the documented data available in the patients’ records regardless of their quality. Conducting similar studies in multiple centers with larger sample sizes should also help clarify our findings. Furthermore, a case-control study design could be used to study the effects of the risk factors in individuals as well as future complications.

## Conclusions

This paper compared risk-factor trends among first-time CABG surgery patients between two different time periods. The most prominent finding in this study is that the percentage of patients who smoked was significantly higher in the second period. This raises concern regarding the insufficient prohibition of tobacco products and water-pipes. To overcome this, both national and individual awareness campaigns and smoking cessation programs should be increased.

## References

[1] Mendis S, Puska P, Norrving B (2020). Global atlas on cardiovascular disease prevention and control: policies, strategies and interventions. https://www.who.int/cardiovascular_diseases/publications/atlas_cvd/en/.

[2] McAloon CJ, Boylan LM, Hamborg T, Stallard N, Osman F, Lim PB. (2016). The changing face of cardiovascular disease 2000-2012: An analysis of the World Health Organisation global health estimates data. Int J Cardiol.

[3] Zeidan RK, Farah R, Chahine MN, Asmar R, Hosseini H, Salameh P Pathak A (2016). Prevalence and correlates of coronary heart disease: first population-based study in Lebanon. Vasc Health Risk Manag.

[4] Al-Daghri NM, Al-Attas OS, Alokail MS (2011). Diabetes mellitus type 2 and other chronic non-communicable diseases in the central region, Saudi Arabia (Riyadh cohort 2): a decade of an epidemic. BMC Med.

[5] Traina MI, Almahmeed W, Edris A, Murat Tuzcu E (2017). Coronary heart disease in the Middle East and North Africa: current status and future goals. Curr Atheroscler Rep.

[6] (2019). Noncommunicable Diseases (NCD) Country Profiles. https://www.who.int/nmh/countries/sau_en.pdf.

[7] oe MT, Chen AY, Cannon CP (2009). Temporal changes in the use of drug-eluting stents for patients with non-ST-Segment-elevation myocardial infarction undergoing percutaneous coronary intervention from 2006 to 2008: results from the can rapid risk stratification of unstable angina patients Supress ADverse outcomes with Early implementation of the ACC/AHA guidelines (CRUSADE) and Acute Coronary Treatment and Intervention Outcomes Network-Get With The Guidelines (ACTION-GWTG) registries. Circ Cardiovasc Qual Outcomes.

[8] Hernandez AF, Li S, Dokholyan RS, O'Brien SM, Ferguson TB, Peterson ED (2009). Variation in perioperative vasoactive therapy in cardiovascular surgical care: data from the Society of Thoracic Surgeons. Am Heart J.

[9] Li Z, Yeo KK, Parker JP, Mahendra G, Young JN, Amsterdam EA (2008). Off-pump coronary artery bypass graft surgery in California, 2003 to 2005. Am Heart J.

[10] Gardner SC, Grunwald GK, Rumsfeld JS (2004). Comparison of short-term mortality risk factors for valve replacement versus coronary artery bypass graft surgery. Ann Thorac Surg.

[11] Gelpi M, Afzal S, Lundgren J (2018). Higher risk of abdominal obesity, elevated low-density lipoprotein cholesterol, and hypertriglyceridemia, but not of hypertension, in people living with human immunodeficiency virus (HIV): results from the Copenhagen comorbidity in HIV infection study. Clin Infect Dis.

[12] O'Donnell MJ, Xavier D, Liu L (2010). Risk factors for ischaemic and intracerebral haemorrhagic stroke in 22 countries (the INTERSTROKE study): a case-control study. Lancet.

[13] Reiner Ž (2014). Primary prevention of cardiovascular disease with statins in the elderly. Curr Atherosclerosis Rep.

[14] Gersh BJ, Sliwa K, Mayosi BM, Yusuf S (2010). Novel therapeutic concepts. The epidemic of cardiovascular disease in the developing world: global implications. Eur Heart J.

[15] Kramarow E, Lubitz J, Francis R Jr (2013). Trends in the coronary heart disease risk profile of middle-aged adults. Ann Epidemiol.

[16] Dadkhah-Tirani H, Hasandokht T, Agostoni P, Salari A, Shad B, Soltanipour S (2018). Comparison of cardiovascular risk factors among coronary artery bypass graft patients in 2010 and 2016: a single-center study in Guilan province, Iran. ARYA Atheroscler.

[17] ElBardissi AW, Aranki SF, Sheng S, O’Brien SM, Greenberg CC, Gammie JS (2012). Trends in isolated coronary artery bypass grafting: an analysis of the Society of Thoracic Surgeons adult cardiac surgery database. J Thorac Cardiovasc Surg.

[18] Dinh DT, Lee GA, Billah B, Smith JA, Shardey GC, Reid CM (2008). Trends in coronary artery bypass graft surgery in Victoria, 2001-2006: findings from the Australasian Society of Cardiac and Thoracic Surgeons database project. Med J Aust.

[19] Clark RE, Edwards FH, Schwartz M (1994). Profile of preoperative characteristics of patients having CABG over the past decade. Ann Thorac Surg.

[20] Ferguson TB Jr, Hammill BG, Peterson ED, DeLong ER, Grover FL; STS National Database Committee (2002). A decade of change—risk profiles and outcomes for isolated coronary artery bypass grafting procedures, 1990-1999: a report from the STS National Database Committee and the Duke Clinical Research Institute. Ann Thorac Surg.

[21] Ziv-Baran T, Mohr R, Yazdchi F, Loberman D (2019). The epidemiology of coronary artery bypass surgery in a community hospital: a comparison between 2 periods. Medicine.

[22] (2019). Defining Adult Overweight and Obesity. https://www.cdc.gov/obesity/adult/defining.html.

[23] Poddar KL, Modi DK, Wayangankar S (2016). Two-decade trends in the prevalence of atherosclerotic risk factors, coronary plaque morphology, and outcomes in adults aged ≤ 45 years undergoing percutaneous coronary intervention. Am J Cardiol.

[24] Chau JY, Grunseit A, Midthjell K, Holmen J, Holmen TL, Bauman AE, Van der Ploeg HP (2015). Sedentary behaviour and risk of mortality from all-causes and cardiometabolic diseases in adults: evidence from the HUNT3 population cohort. Br J Sports Med.

[25] Proper KI, Singh AS, Van Mechelen W, Chinapaw MJ (2011). Sedentary behaviors and health outcomes among adults: a systematic review of prospective studies. Am J Prev Med.

[26] Aljefree N, Ahmed F (2015). Prevalence of cardiovascular disease and associated risk factors among adult population in the Gulf region: a systematic review. Adv Public Health.

[27] Kasliwal R, Kulshreshtha A, Agrawal S, Bansal M, Trehan N (2006). Prevalence of cardiovascular risk factors in Indian patients undergoing coronary artery bypass surgery. JAPI.

[28] Ji Q, Zhao H, Mei Y, Shi Y, Ma R, Ding W (2015). Impact of smoking on early clinical outcomes in patients undergoing coronary artery bypass grafting surgery. J Cardiothoracic Surg.

[29] Etemadi A, Khademi H, Kamangar F (2017). Hazards of cigarettes, smokeless tobacco and waterpipe in a Middle Eastern population: a cohort study of 50 000 individuals from Iran. Tobacco Control.

[30] Cornwell LD, Omer S, Rosengart T, Holman WL, Bakaeen FG (2015). Changes over time in risk profiles of patients who undergo coronary artery bypass graft surgery: the Veterans Affairs Surgical Quality Improvement Program (VASQIP). JAMA Surgery.

